# Supplementary material for: Direct Oral Anticoagulants Are Associated with Superior Survival Outcomes than Warfarin in Patients with Head and Neck Cancers
Source: Cancers (Basel). 2022 Jan 29;14(3):703. doi: 10.3390/cancers14030703 (PMC8833638; doi:10.3390/cancers14030703)
Supplement: Supplementary file 1 [file cancers-14-00703-s001.zip › cancers-1549890 - sup.pdf]

Table S1. Dose and duration of DOACs and Warfarin.

Anticoagulants	N	Dose(mg)		Duration (days)	
		Mean(±SD)	Median(IQR)	Mean(±SD)	Median(IQR)
**DOACs**					
Apixaban	21	8.21(±2.64)	10.0(5.0-10.0)	226(±196.2)	184(56-352)
Dabigatran	8	256.25(±69.27)	300(220-300)	307.8(±168.8)	352.5(161.0-401.5)
Edoxaban	11	46.36(±15.67)	60(30-60)	226.6(±140.8)	217(119-313)
Rivaroxaban	52	17.55(±7.37)	15(10-20)	272.1(±270.1)	161.0(70.0-429.5)
**Warfarin**	113	2.54(±1.45)	2.5(2.0-2.5)	376.9(±627.0)	168(56-350)

We analyzed the effects of four different DOACs on the overall survival (OS) and disease-specific survival (DSS) of head and neck cancer patients. There were 21 patients using Apixaban, 8 using Dabigatran, 11 using Edoxaban and 52 patients using Rivaroxaban. No significant differences were found in either OS (p = 0.4361, Figure S1) or DSS (p = 0.3602, Figure S2) among these four different DOAC users, which could be due to the small sample size.

In addition, we also compared the users of these four different DOACs with warfarin users and non-users for survival benefits. Significant difference was found in OS (p = 0.0234, Figure S3) but not in DSS (p = 0.2006, Figure S4).

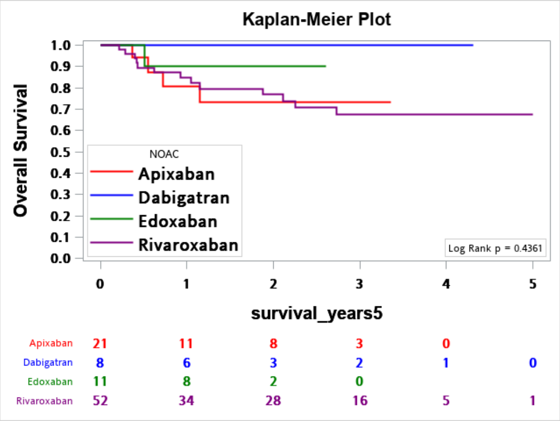

Figure S1. Overall survival between Apixaban users (n=21), Dabigatran users (n=8), Edoxaban users (n=11), and Rivaroxaban users (n=52).

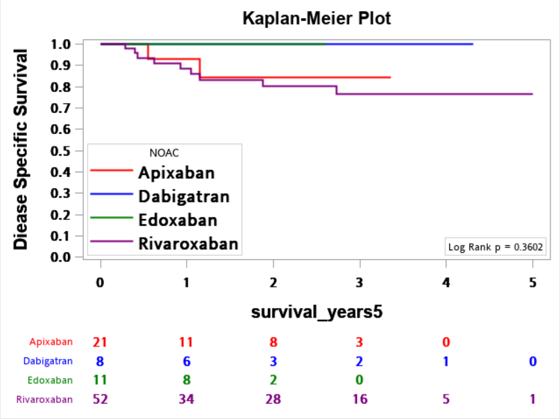

Figure S2. Disease specific survival between Apixaban users (n=21), Dabigatran users (n=8), Edoxaban users (n=11), and Rivaroxaban users (n=52).

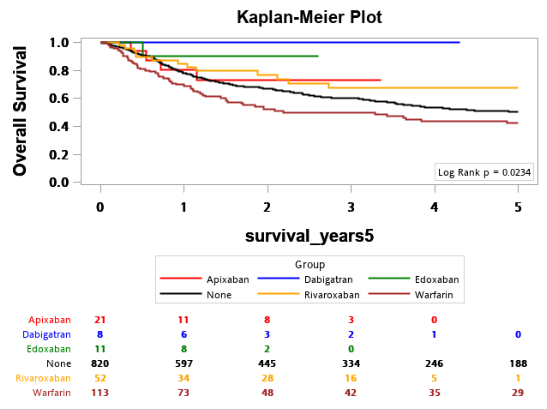

Figure S3. Overall survival between Apixaban users (n=21), Dabigatran users (n=8), Edoxaban users (n=11), Rivaroxaban users (n=52), Warfarin users (n=113), and none-users (n=820)

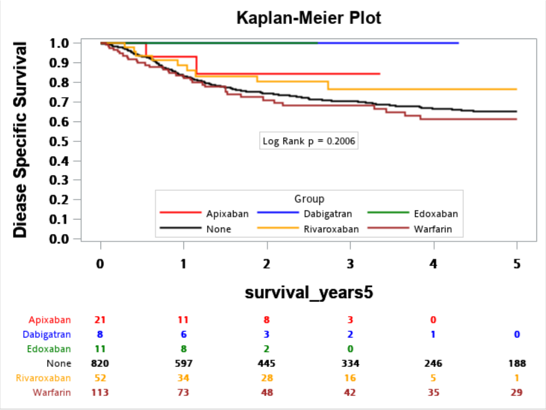

Figure S4. Disease specific survival between Apixaban users (n=21), Dabigatran users (n=8), Edoxaban users (n=11), Rivaroxaban users (n=52), Warfarin users (n=113), and none-users (n=820).
